# Supplementary material for: Neural Responses to a Working Memory Task in Acute Depressed and Remitted Phases in Bipolar Patients
Source: Brain Sci. 2023 Apr 29;13(5):744. doi: 10.3390/brainsci13050744 (PMC10216136; doi:10.3390/brainsci13050744)
Supplement: Supplementary file 1 [file brainsci-13-00744-s001.zip › brainsci-2312665-SI.pdf]

Supplementary Table 1: Table of medications of remitted BD patients in both phases of disorder:

	Remitted/A BD (n = 15)	Remitted/R BD (n = 15)
Antipsychotic	12 (80%)	8 (53%)
More than one antipsychotic	1 (6%)	1 (6%)
Lithium	9 (60%)	9 (60%)
SSRI	6 (40%)	6 (40%)
Tricyclic antidepressive	4 (26%)	6 (40%)

BD – Bipolar Disorder Patients; Remitted/A – for remitted patients on acute phase; Remitted/R – for remitted patients on remitted phase; SSRI - Selective Serotonin Reuptake Inhibitor. Data present in: N (% relative to N of their respective group).

Supplementary Table 2: Table of medications of remitted BD patients compared to acute phase:

	**Medication Dose difference from Acute to Remitted phase (n = 15)**			This is a new medication	Took off this medication
	Decreased	Maintained	Increased		
Antipsychotic	5 (62%)	1 (12%)	2 (25%)	-	4 (50%)
Lithium	3 (33%)	-	4 (66%)	2 (22%)	1 (11%)
SSRI	-	4 (66%)	1 (16%)	1 (16%)	1 (16%)
Tricyclic antidepressive	-	1 (16%)	1 (16%)	4 (66%)	2 (32%)

SSRI - Selective Serotonin Reuptake Inhibitor. Data present in: N (% relative to the N of this medication class on remitted phase).

Description of the sample:

Thirty-seven patients with bipolar disorderBD in their depressive phase were recruited. One of these patients had to be excluded due to poor task performance. Patients were recruited while being inpatients in a specialized bipolar ward at the Department of Psychiatry, Psychosomatics and Psychotherapy of the University Hospital Würzburg. Patients have been diagnosed along DSM-IV criteria by two trained psychiatrists and diagnosis was confirmed by the OPCRIT diagnostic system (McGuffin, Farmer, & Harvey, 1991). Patients were measured in a depressed phase according to DSM-IV (Montgomery Asperger Depression Rating Scale [MADRS] scores (Montgomery & Asberg, 1979): mean = 14.6 ± 10.3; Young Mania Rating Scale [YMRS] scores to exclude mixed episodes (Young, Biggs, Ziegler, & Meyer, 1978): mean = 3.4 ± 3.7). Fifteen patients could be enrolled for a follow-up measurement at least three months later and after reaching remission, which had been established by the treating psychiatrist and confirmed in semi-structured interviews (MADRS mean = 3.5 ± 1.5; YMRS mean = 1.1 ± 1.3). Medication was recorded to assesscontrol for possible confounding effects of the results with medication. Twenty-four patients had antipsychotic medication, 8 patients took more than one antipsychotic medication. Eighteen patients took lithium, fourteen patients took an SSRI, and eight patients had tricyclic antidepressive medication. No patient was medication free, no patient had a medication monotherapy. To obtain the healthy control group, flyer were put out around the town of Würzburg, an add in the paper was also used. People who were interested in participating were screened for age, education level and psychiatric disorders. Controls were matched to the total sample of patients in age, sex and performance on the multiple-choice word test (MWT-B) (Lehrl, 2005), which measures crystallized intelligence. All controls were free of past and current axis I disorders (classified via the DSM-IV) as assessed with the Mini International Neuropsychiatric Interview (MINI), German Version 5.0 (Sheehan et al., 1998).

Supplementary Table 3: Demographics of the respective test subjects. In the test statistics the first statistic in a row is always the comparison between depressed patients and controls, the second statistic indicates the comparison between remitted patients and controls. Depressed and remitted patients were not compared since they are identical.

	Depressed Bipolar Patients	Remitted Bipolar Patients	Healthy Controls	
Sex ♂ / ♀	19/ 17	10/5	10/20	Chi2 =2.5 , p = .1<br>Chi2 =4.5 , p = .03
Age	42 ± 11.2	36 ± 11	42.3 ± 10.7	T(64)=.14, p=.9<br>T(43)=1.9, p=.07
MWT-B	29.2 ± 4.9	30.6 ± 3.8	29 ± 8.6	T(64)=.13, p=.9<br>T(40)=.67, p=.5
